# µgreen-db: a reference database for the 23S rRNA gene of eukaryotic plastids and cyanobacteria

**DOI:** 10.1038/s41598-020-62555-1

**Published:** 2020-04-03

**Authors:** Christophe Djemiel, Damien Plassard, Sébastien Terrat, Olivier Crouzet, Joana Sauze, Samuel Mondy, Virginie Nowak, Lisa Wingate, Jérôme Ogée, Pierre-Alain Maron

**Affiliations:** 1 Agroécologie, AgroSup Dijon, INRA, University Bourgogne Franche-Comté, Dijon, France; 2 Plateforme GenomEast, IGBMC, CNRS UMR7104 Illkirch, France; 3 Univ. Paris Saclay, AgroParisTech, UMR ECOSYS, INRA, F-78206 Versailles, France; 4 INRA, Bordeaux Science Agro, UMR 1391 ISPA, 33140 Villenave d’Ornon, France

**Keywords:** Computational biology and bioinformatics, Ecology, Microbiology, Molecular biology, Ecology

## Abstract

Studying the ecology of photosynthetic microeukaryotes and prokaryotic cyanobacterial communities requires molecular tools to complement morphological observations. These tools rely on specific genetic markers and require the development of specialised databases to achieve taxonomic assignment. We set up a reference database, called µgreen-db, for the 23S rRNA gene. The sequences were retrieved from generalist (NCBI, SILVA) or Comparative RNA Web (CRW) databases, in addition to a more original approach involving recursive BLAST searches to obtain the best possible sequence recovery. At present, µgreen-db includes 2,326 23S rRNA sequences belonging to both eukaryotes and prokaryotes encompassing 442 unique genera and 736 species of photosynthetic microeukaryotes, cyanobacteria and non-vascular land plants based on the NCBI and AlgaeBase taxonomy. When PR^2^/SILVA taxonomy is used instead, µgreen-db contains 2,217 sequences (399 unique genera and 696 unique species). Using µgreen-db, we were able to assign 96% of the sequences of the V domain of the 23S rRNA gene obtained by metabarcoding after amplification from soil DNA at the genus level, highlighting good coverage of the database. µgreen-db is accessible at http://microgreen-23sdatabase.ea.inra.fr.

## Introduction

Photosynthetic microeukaryotes and cyanobacteria can be found in diverse aquatic and terrestrial habitats thanks to their advanced abilities to adapt to a range of challenging conditions, including extreme environments such as polar regions or deserts (*e.g*., soils, marine water, freshwater and brackish water, air, plants, and animals)^1–5^. These ubiquitous microorganisms play essential ecological roles in the global carbon and nitrogen cycles and also contribute to the production of atmospheric oxygen. As primary producers, they form the base of trophic chains (*e.g*., microbial loops in aquatic ecosystems^6^) and may represent a potentially rich reservoir for diverse, natural biosynthetic products^7^.

Soil photosynthetic microbes primarily belong to three main groups: prokaryotic cyanobacteria, and two groups of photosynthetic microeukaryotes including green algae and diatoms^8–10^. Cyanobacteria play a major role in the evolution of terrestrial ecosystems through their involvement in oxygenic photosynthesis. Cyanobacteria can be used as a source of biofertiliser because they fix atmospheric nitrogen and thereby improve the sustainability of agriculture^11^. Green algae (Chlorophyta) are components of desert soil communities and can live in symbiotic association with fungi or cyanobacteria; they contribute to nutrient cycling^12^. Terrestrial diatom ecology has been poorly investigated to date but appears to be promising as a soil quality indicator such as a tracer of hydrological processes^13^ or to evaluate heavy metal contamination in soils^14^. Photosynthetic microeukaryotes represent a polyphyletic assemblage including several lineages that evolved from a common primary endosymbiosis: the main group of green algae (Viridiplantae) belongs to a well-supported monophyletic group subdivided in two major groups, namely Chlorophyta and Streptophyta [this second group includes Charophyta and land plants, red algae (Rhodophyta, also known as Rhodophyceae) and glaucophytes (Glaucophyta)]. Other protozoa groups such as euglenids belonging to Excavata (Euglenozoa), Cercozoa belonging to Rhizaria, and Chromista groups such as cryptomonads (Cryptophyta), haptophytes (Haptophyta or brown algae), stramenopiles [Bacillariophyta (or diatoms) and Ochrophyta)], and Dinoflagellates (Miozoa, also known as Myzozoa) have a secondary endosymbiotic origin^15–20^.

The diversity and composition of the microbial photosynthetic community can be used as a bioindicator of soil quality^21^ and of the presence of invasive species. Microbial photosynthetic communities can also help identify and monitor the involvement of specific groups in the biodegradation of environmental pollutants^5,20,22^. In addition, a better understanding of microbial photosynthetic community diversity can help understand their function and contribution to C cycling, notably in marine^23^ and dryland^24^ ecosystems.

A large body of knowledge on the taxonomy of photosynthetic microeukaryotes and cyanobacteria gathered from microscopic observations during the past century. However, in the past twenty years, phylogenetic analyses have demonstrated that an approach based on morphological determination alone is somewhat artificial for most of the microalgal genera and should be revised^25,26^. Several studies recently estimated the diversity of indigenous photosynthetic microbial communities in various environments using metabarcoding coupled with high-throughput sequencing (HTS)^3,27–34^. A range of molecular markers has been used to describe cyanobacterial and photosynthetic microeukaryote diversity with varying degrees of resolution (*e.g*., 16S/18S/23S rRNA, *tuf*A, *psbA*, rbcL, ITS)^32,35–39^. Various hypervariable regions (*e.g*., V4, V8–V9) of the 18S rRNA gene are commonly used^40^. However, the 23S rRNA gene presents several advantages over the other markers. In particular its length and higher sequence variability provide a better phylogenetic resolution than small rRNA subunits^41,42^. More precisely, domain V of the 23S rRNA gene, known as the universal plastid amplicon (UPA), allows the targeting of organisms containing plastids with a remarkable universality, covering most photosynthetic microbial groups^43,44^. For cyanobacteria, this marker also seems to be promising as it provides better coverage of community diversity than 16S rDNA or *tuf*A^37^. Moreover, UPA has a length (~410 bp) suitable for HTS Technologies^43^, such as Illumina^45^. UPA can also be used in addition to other markers to obtain a comprehensive overview of microbial diversity^31,37,39,46^.

Major collaborative projects and studies at the international level (*e.g*., UniEuk, EukRef) are currently underway to propose a classification of microbial eukaryotes that will serve as a reference for a universal taxonomy^47–49^. The proposed tools are mainly deployed on the 18S rRNA gene that targets eukaryotic photosynthetic organisms but does not target cyanobacteria.

Metabarcoding still remains the fastest and cheapest method to study microbial diversity and community structure. However, it requires reference databases, updated with a good coverage of organisms, a high level of sequence quality, and curated taxonomy to achieve the taxonomic assignment of the retrieved sequences^50^. There already exist several generalist or specialist databases that include groups of photosynthetic microeukaryotes and cyanobacteria with curated taxonomy. The most popular databases are (i) SILVA, that groups SSU and LSU rRNA genes from eukaryotic and prokaryotic organisms^51^, (ii) PR^2^, a protist small subunit ribosomal reference database^52^, (iii) PhytoREF, a reference database of the plastidial 16S rRNA gene of photosynthetic eukaryotes^53^, (iv) R-Syst::diatom, that gathers the 18S rRNA gene and rbcL diatom sequences^54^, and (v) DINOREF, a reference database of the 18S rRNA of dinoflagellates^55^. Sherwood *et al*.^31^ recently made available a database (http://scholarspace.manoa.hawaii.edu/handle/10125/42782) that groups 97,194 UPA and LSU amplicon sequences from their own project, including sequences not found in SILVA. However, these sequences are mainly assigned to the Bacteria domain (75% of the total sequences with only <1% assigned to Cyanobacteria), whilst within the 10% of eukaryotic sequences, 80% are associated to the Metazoa group. Moreover, the taxonomy is not fully standardised and therefore difficult to use for HTS analyses. A reference database of the UPA marker exists; it only contains taxa related to photosynthetic microeukaryotes and cyanobacteria, as well as standardized taxonomy. However, it includes far fewer sequences (573) than the other UPA databases described above^56^. Thus, to our knowledge, no 23S rRNA database exists to date that meets all the essential criteria (*i.e*., good coverage of organisms, good sequence quality, curated taxonomy) for the molecular study of photosynthetic microeukaryote and cyanobacterial communities.

We propose a new reference database of 23S rRNA gene sequences in eukaryotes and cyanobacteria, called µgreen-db. It was constructed from various sources (SILVA, CRW, BLAST, or sequences extracted from genomes) so as to be the most representative one available. When possible, the complete sequence of the 23S rRNA gene is provided, allowing users to create their own primers for environmental metabarcoding studies. The overall taxonomy associated with the sequences is based on the PR^2^/SILVA, or NCBI or AlgaeBase databases. In µgreen-db, sequences of non-vascular land plants are also provided to improve the study of photosynthetic microeukaryote and cyanobacterial communities in soil environments where mosses and liverworts (Bryophytes) can be abundant. Thus, the inclusion of sequences related to bryophyte taxa will help avoid orphan sequences and improve the recovery of taxonomic information from sequence datasets. This database is open-source and can be downloaded from the website http://microgreen-23sdatabase.ea.inra.fr.

## Results

### Overview of µgreen-db

**Figure 1:**
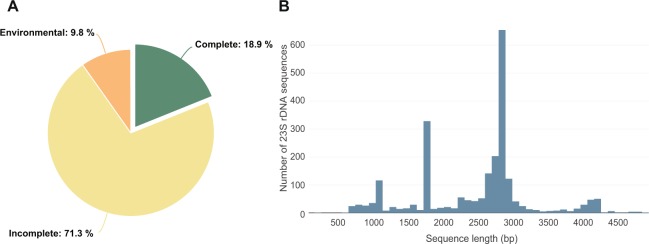
Pie chart and histograms showing (**A**) the origin and number, and (**B**) the length of the 23S rDNA sequences available in the database.

µgreen-db currently contains 2,326 non-redundant sequences including 440 complete, 1,658 incomplete, and 228 environmental 23S rDNA sequences (Fig. 1A). Two thousand, two hundred and seventy-one sequences are between 800 and 4,000 bp in length (Fig. 1B).

µgreen-db provides a reference file containing all the sequences in fasta format. For each sequence, the associated identifier is in the following form: [C or I or E]AccessionNumber.Letter(if duplicate).start.end;AllLineage where ‘C’ means complete, ‘I’ incomplete and ‘E’ environmental. We also provide a set of two files, a reference sequence file with a unique identifier in fasta format and another with the complete taxonomy that can be used easily in the most popular metabarcoding pipelines (Mothur, QIIIME, GnS-PIPE, DADA2) with NCBI, or AlgaeBase or PR^2^/SILVA taxonomies.

### Taxonomic validation – taxonomic composition of μgreen-db

Following the initial retrieval of the database sequences in June 2016, a further update of the entire taxonomy was completed using NCBI in August 2018. During this update we encountered three scenarios for each sequence, *i.e*., (i) no change in taxonomy, (ii) obsolete accession number (8 sequences), or (iii) removal or loss of the accession number (2 sequences). In the case of (ii), we updated the accession number, while in the case of (iii), we removed these particular sequences from our database.

**Figure 2:**
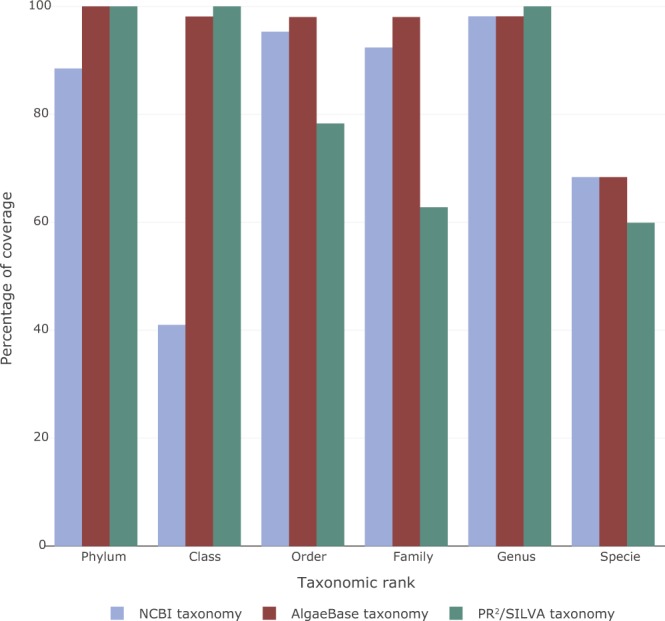
Taxonomic coverage at different ranks from the PR^2^/SILVA, NCBI and AlgaeBase taxonomy.

Taxonomic coverage (corresponding to the percentage of sequences for a given rank) was higher with AlgaeBase than with NCBI (Fig. 2).

**Figure 3:**
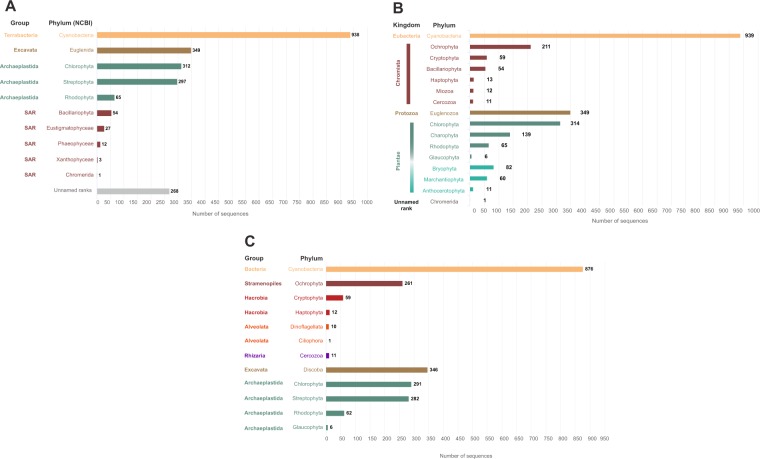
Sequence distribution of the µgreen-db database at the Phylum level and grouped by Kingdom or supergroups. (**A**) Based on NCBI taxonomy according to Adl *et al*. (2012)^55^ for the group classification, (**B**) Based on AlgaeBase taxonomy, (**C**) Based on PR^2^ and SILVA taxonomy.

Coverage at the class and genus levels was slightly better with PR^2^/SILVA than with AlgaeBase. For sequences assigned from the NCBI database, we obtained 88.5% and 42% coverage at the phylum and class ranks, respectively (Fig. 2). We obtained 10 phyla across the 4 supergroups (Terrabacteria, Excavata, Archaeplastida, and SAR), but 11.5% of the sequences were had no taxonomic assignment at this phyla rank (Fig. 3A).

When we used AlgaeBase taxonomy for the sequence assignment, we obtained 100% coverage at the phylum level (Fig. 2), with 1 phylum for the Eubacteria kingdom, 6 phyla for the Chromista kingdom, 1 for the Protozoa kingdom, and 7 for the Plantae kingdom; 4 of them were photosynthetic microeukaryotes, and 1 Chromerida phylum had no kingdom affiliation (Fig. 3B). The most represented phylum was Cyanobacteria with 939 sequences, followed by Euglenozoa (349 sequences), and Chlorophyta (314 sequences), while Bacillariophyta was less represented (54 sequences) (Fig. 3B).

Two thousand, two hundred and eighty-three sequences (*i.e*., 98% of the total sequences) were assigned up to the genus rank (442 unique genera) by NCBI and AlgaeBase taxonomies; the top 3 of the most represented genera were Prochlorococcus (207 species), Chroococcidiopsis (120), and Synechococcus (90), all belonging to cyanobacteria. A total of 1,590 sequences were affiliated at the species level, including 736 unique species, by NCBI and AlgaeBase taxonomies (not including sequences identified as “uncultured” or sequences non-affiliated at the species level, *i.e*., .sp).

µgreen-db based on PR^2^/SILVA taxonomy contained 2,217 of the 2,326 retrieved sequences, distributed across seven groups (Bacteria, Stramenopiles, Hacrobia, Alveolata, Rhizaria, Excavata, Archaeplastida) (Fig. 3C), with 399 unique genera and 696 unique species available (with the same top 3 as previously) as well as 93.3% of Cyanobacteria, 97% of photosynthetic microeukaryotes, and 92.8% of non-vascular land plants.

To finalise our database, we used the universal primer pair to amplify the 23S rRNA V region (UPA)^44,57^ in our database. We obtained 1,500 of the 2,366 sequences with a PCR *in silico* (Supplementary Fig. S1). Several formatted files were generated for metabarcoding data analysis (https://zenodo.org/record/3385760#.XW-NptPVLUI).

### Description of the µgreen-db web interface

µgreen-db is also available *via* a web interface (http://microgreen-23sdatabase.ea.inra.fr). Access to all data is provided *via* this interface and simply allows searches for taxa of interest. Using this website, one can also download the latest sequence files with NCBI-, AlgaeBase- or PR^2^/SILVA-based taxonomies in various formats compatible with the commonly used bioinformatic pipelines. Finally, information on the construction of this database, statistics and news are also accessible through this website.

### Illustration of the application of µgreen-db for the study of complex soil phototrophic microbial communities

**Figure 4:**
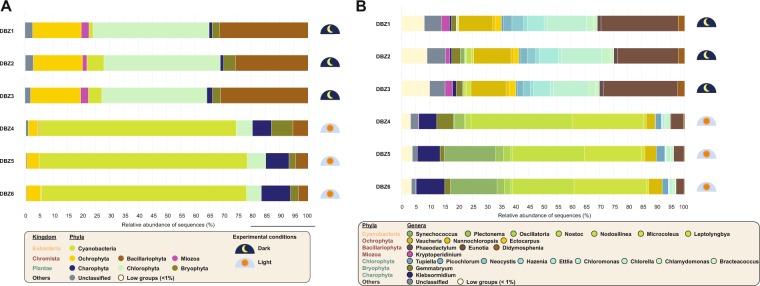
Relative sequence abundance of photosynthetic microeukaryotes and cyanobacteria at Phylum (**A**) and Genus (**B**) level.

We tested the ability of µgreen-db to assign sequence datasets generated from a set of indigenous soil phototrophic microbial communities obtained from a soil exposed to two contrasted light conditions (dark *vs*. light). The Shannon diversity indices calculated from the OTU dataset highlighted higher diversity under the dark condition than under the light condition (*H’* = 3.1 ± 0.1 *vs. H’* = 2.6 ± 0.1, respectively) (Table S1). This decrease in diversity was associated to a lower richness (441.3 ± 41.2 *vs*. 378.3 ± 31.4 OTUs) and a lower evenness (0.51 ± 0.01 *vs*. 0.43 ± 0.02) of the community after exposure to light. Interestingly, µgreen-db correctly affiliated 98.5% and 96% of the sequence datasets at the phylum and genus levels, respectively. The taxonomic affiliation of the sequences also revealed a broad diversity of the phototrophic soil microbial community, with 11 phyla and 149 unique genera detected. As observed for the diversity metrics, light conditions significantly shaped the composition of the phototrophic community. Most markedly, Cyanobacteria became highly dominant at the phylum level, increasing from 4 ± 2.4% to 72.0 ± 1.8% of the assigned sequences after exposing the soil to light (Fig. 4A).

In the same way, sequences related to Charophyta increased from 1.4 ± 0.5 to 8.4 ± 1.8% following exposure to light. In contrast, Chlorophyta, Bacillariophyta and Ochrophyta, which represented 39.75 ± 2.37%, 29.2 ± 3.1%, and 17.4 ± 0.2% of the sequences in the dark treatment, decreased to 5.9 ± 0.7, 4.4 ± 0.9 and 4.3 ± 0.8%, respectively after light exposure (Fig. 4A). Furthermore, the Miozoa phylum disappeared under the light treatment. All phyla were typically and consistently found in all three sample replicates, except Anthocerotophyta that was detected in only one of the three replicates of the light treatment (Fig. 4A). The clear taxonomic separation of the dark and light treatments was also observed at the genus level (Fig. 4B). The increase in Cyanobacteria under light was mainly caused by the stimulation of three genera: *Microcoleus*, *Nodosilinea* and *Synechococcus*. *Klebsormidium* was the only genus that explained the increase in Charophyta in response to light. In contrast, there was a higher contribution of Chlorophyta, Bacillariophyta and Ochrophyta in the dark treatment caused by the higher occurrence of genera such as *Chlorella* and *Ettlia* (Chlorophyta), *Eunotia* (Bacillariophyta) and *Ectocarpus*, *Nannochloropsis* and *Vaucheria* (Ochrophyta).

## Discussion

Microbial diversity can be studied using either morphological identification or molecular tools that assign taxa based on genetic markers. Several authors recommended to combine these two methods to obtain improved coverage of species^37,58,59^. This combination of techniques is particularly powerful to improve our functional understanding of photosynthetic microbial communities.

As stated before, the µgreen database contains sequences retrieved from very diverse sources/methodologies and offers the possibility to affiliate sequences based on three different taxonomy nomenclatures: PR^2^/SILVA, NCBI, and AlgaeBase. To allow for an efficient taxonomic assignment, we provide full lineage from the kingdom/phylum levels down to the species level. Nevertheless, μgreen-db is not a phylogenetic or taxonomic authority. One limitation in using the 23S rRNA gene in metabarcoding studies is that few sequences are available from public databases (*e.g*., GenBank, SILVA)^57,60^. This explains why it was necessary to retrieve our sequences using several strategies to obtain the most diverse database possible. In addition, to retrieve the sequences from various databases, we implemented a strategy of recursive BLAST with phylogenetic tree construction to improve our spectrum of organisms. Consequently, we were able to recover more than 1,500 sequences and to significantly increase the total number of sequences in our reference database.

The taxonomic assignment of sequences by NCBI provided contrasting results. Although taxonomy for almost all sequences were assigned at the genus level, only 88.5% of them were assigned at the phylum level, and 42% at the class level. The low percentage at class level comes in part from the incomplete taxonomy of cyanobacteria in NCBI. Indeed, almost all of the sequences assigned to cyanobacteria (933/938) do not have a class name in NCBI database. The other sequences with an unnamed rank at the class level (440/1373) are distributed across different phyla in photosynthetic microeukaryotes. We also noticed diverging rankings between the PR^2^/SILVA, NCBI and AlgaeBase databases. For example, the cryptomonads group was ranked at the class level by NCBI and at the phylum level by AlgaeBase. The classification of this particular group remains widely debated; this is why we chose to propose both affiliations, and leave it up to the user to decide. Indeed, it was not until very recently that the consensus classification for eukaryotes^61^ started using any ranks at all. Thus, cryptomonads are just listed as ‘Cryptophyceae’ (not the phylum/division Cryptophyceae or the class Cryptophyceae) but should now be classified down to the order level^43^. Another example is the Phaeophyceae group that is associated at the phylum level by NCBI but at the class level by AlgaeBase. As stated on the NCBI website, their taxonomy database is not an authoritative source for nomenclature or classification. For this reason, we recommend using AlgaeBase taxonomy because it provides manual curation, and offers a very complete bibliography for each taxon^61,62^.

Analysis of the environmental soil samples validated the power of µgreen-db to characterise the taxonomic composition of indigenous phototrophic microbial communities. We were able to assign 98.5% of the sequences at the phylum level and 96% at the genus level, highlighting good coverage of phototrophic diversity in the database based on AlgaeBase taxonomy. From a biological point of view, our results provided evidence for a strong impact of the photoperiod on the composition and diversity of the phototrophic microbial community.

Under long-term dark incubation, the dominant photosynthetic microeukaryotes were related to species with a mixotrophic strategy to remain active in the dark, and/or to species better able to overcome unfavourable light conditions by switching to dormant forms and/or producing resistant forms. A number of the photosynthetic microeukaryote taxa detected in the dark-conditioned soil across the dominant phyla (Chlorophyta, Bacillariophyta and Ochrophyta) can modulate their metabolism from phototrophic to heterotrophic by assimilating dissolved organic carbon depending on prevalent environmental conditions^63,64^. Such trophic and flexible metabolic strategies are an important competitive advantage in soils, where light can rapidly become a limiting factor for obligate autotrophs^65^ during photosynthetic growth, as reported in lakes^66^. In our study, the dominance of some photosynthetic microeukaryote classes under continuous dark conditions stressed that they may be equally adapted to survive using obligate chemoheterotrophic metabolism. In contrast, Cyanobacteria with limited mixotrophic capacities may not be equally able to grow efficiently using chemoheterotrophy over long periods of time^67^. Moreover, the relatively strong occurrence of certain species (*e.g., Vaucheriaceae*) currently not considered as mixotrophs^68^ may result from the ability of these organisms to switch to a dormant stage under unfavourable conditions and produce resistant forms (zygospores, akinetes, zoospores). Such forms of resistance or dispersal stages have been reported for a wide range of Cyanobacteria and photosynthetic microeukaryotes^69^.

During the light treatment, the strong development of numerous cyanobacterial taxa over-competing photosynthetic microeukaryotes might also be partially explained by the high soil alkalinity (pH = 8.2). Alkaline soils are known to promote cyanobacteria over eukaryotic green algae^70,71^. Under our experimental conditions (optimum water content, temperature and light), cyanobacteria may have been favoured because they have relatively faster growing strategies with shorter generation times than photosynthetic microeukaryotes. This could explain why the soil surface became overrun by cyanobacteria and contributed to the lower diversity indices observed under light conditions. µgreen-db now paves the way for future studies investigating the community and functional ecology of photosynthetic organisms in soils.

In conclusion, our results demonstrate that µgreen-db is a powerful tool to assign the 23S rRNA genes of photosynthetic microeukaryotes and cyanobacteria of soil environments to different taxonomic levels. Future improvements to the database will consist in (i) setting up regular routines (once a year) to enrich this open-access database by adding new sequences (*e.g*., SILVA r132), and (ii) assimilating any accession changes by updating NCBI accession numbers and taxonomy from various sources. We also encourage the future community of users to contact the curators of the database to report any errors found in the database or on the website, or *via* the website portal or directly by email to the corresponding author.

## Methods

### Retrieval of 23S rDNA sequences from public databases

**Figure 5:**
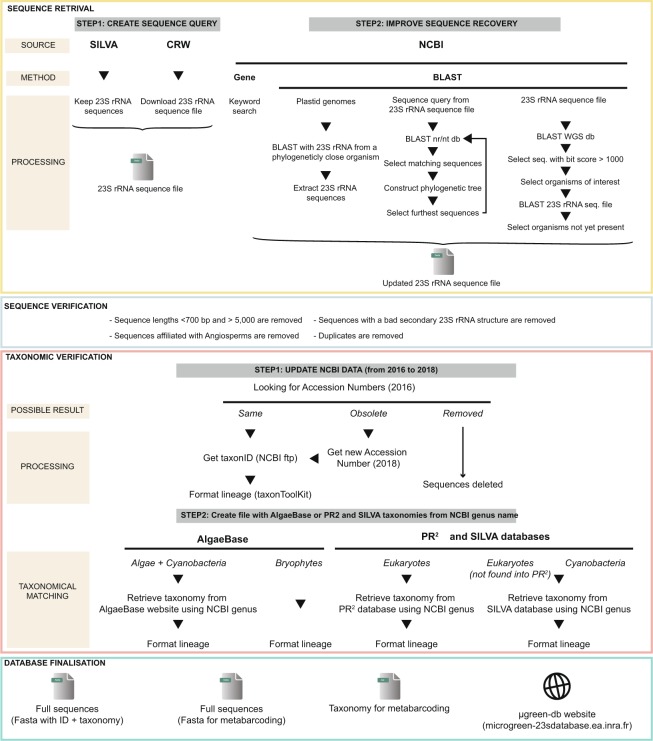
Workflow describing the different steps performed to generate the curated and annotated 23S rDNA reference database constructed from various databases and methods.

We developed several strategies to recover the maximum number and diversity of sequences possible (Fig. 5).

23S rRNA sequences in cyanobacteria, photosynthetic microeukaryotes and bryophytes were retrieved from SILVA r123 (June 2016)^51^. We also retrieved 23S chloroplast sequences from various organisms (photosynthetic microeukaryotes, bryophytes, angiosperms) from a Comparative RNA Web Site and Project led by the Gutell Lab at the University of Texas (Austin, USA) (www.rna.ccbb.utexas.edu/DAT/3C/Alignment/)^72^. Another set of sequences was also recovered from NCBI with the Gene database (the list of different queries is available in Supp. data file 1). We also used various BLAST (with a megablast approach with a maximum target parameter of 1,000) to improve sequence recovery. We first performed a BLAST with a 23S rRNA sequence from a phylogenetically close organism on plastid genomes. We then performed a second BLAST by taking a sequence query in the nr/nt database and retrieved all the returned sequences. Based on these sequences, we built a phylogenetic tree recursively to know which sequence was furthest away every time. Then we aligned the sequences using Muscle (Mega7)^73^ and reconstructed the phylogenetic tree using a maximum-likelihood method^74^. To improve µgreen-db exhaustivity, we performed another BLAST against the NCBI WGS database, selecting sequences with a bit score greater than 1,000 and belonging to the targeted organisms, and performed a final BLAST from these sequences against the 23S rRNA sequence file. Sequences corresponding to taxa not present in the 23S rRNA sequence file were then selected and added to the sequence dataset based on less than 97% identity to increase the diversity of the sequences affiliated at the genus level in µgreen-db. For all BLAST strategies we did not use the “mask” option at all and we requested BLAST to perform a global alignment of the retrieved sequences with the reference sequences. Only sequences presenting the best alignment score (based on percent of identity; >97% to find new species and <97% to find new genera or families) were finally kept. This minimised the possible bias of BLAST alignment due to the variability of sequence lengths in the dataset, with sequences possibly including conserved and/or variable regions.

### Sequence verification

According to the origins of the sequences, we applied a series of different filters to retain only plastid sequences (Fig. 5). Regarding SILVA sequences, we only kept sequences with a length higher than 700 bp and a quality ≥75%. For the sequences recovered from other databases, we also verified the secondary structure, using the INFERNAL tool^75^. Finally, we checked the non-redundancy of the sequences to retain only unique sequences. For each sequence found in both the SILVA and BLAST databases, we checked whether the sequence was included in the ‘BLAST’ sequence *(i.e*., at the identity level). If such was not the case, we aligned them and kept the least fragmented sequence. We also removed the sequences assigned to Angiosperms from the CRW database (Supplementary Fig. S2). Long sequences (more than 5,000 bp) were also deleted.

### Taxonomic validation – The taxonomic framework of µgreen-db

PR^2^/SILVA, NCBI and AlgaeBase taxonomies were all retrieved to provide users the choice for further analyses (Fig. 5). To obtain a standardised taxonomy in the form of phylum, class, order, family, genus and species, we recovered the *taxonID* from the NCBI accession number (ftp://ftp.ncbi.nih.gov/pub/taxonomy/accession2taxid/) and used the *taxonkit* tool (http://github.com/shenwei356/taxonkit) to retrieve the full lineage. AlgaeBase taxonomy was also used to obtain more information at the kingdom level. When no ranking information was available, we ascribed the abbreviation rank followed by two underscores plus Unnamed_rank (*e.g*., p__Unnamed_rank). As non-vascular land plants are not represented in AlgaeBase, we assigned the Plantae Kingdom from NCBI taxonomy to these sequences and made modifications at the phylum level. All these sequences were assigned to the phylum Streptophyta by NCBI taxonomy. However, as Streptophyta is an infrakingdom subdivided into three phyla in AlgaeBase, we assigned the classes Bryopsida, Polytrichopsida, Sphagnopsida, Tetraphidopsida, Takakiopsida, Andreaeobryopsida, Andreaeopsida, Oedipodiopsida to the Bryophyta phylum, Jungermanniopsida, Marchantiopsida, Haplomitriopsida to the Marchantiophyta phylum, and Anthocerotopsida and Leiosporocerotopsida to the Anthocerotophyta phylum. To format the PR^2^/SILVA taxonomy, the full lineage was constructed with the NCBI genus name by searching the PR^2^ database (https://github.com/pr2database/pr2database) and SILVA taxonomy archive (https://www.arb-silva.de/no_cache/download/archive/current/Exports/taxonomy/).

### Database finalisation – construction of the µgreen-db database

The database is available in two forms: from tabular flat files, and from a website (http://microgreen-23sdatabase.ea.inra.fr) (Fig. 5). The tabular flat files were formatted with a custom homemade script. The web interface was built using Bulma (https://bulma.io), a modern and open-source CSS framework based on Flexbox with a custom template. The website uses PHP (v7.2.7) to communicate with the MySQL database, providing back-end storage of sequences and taxonomy by using queries and Javascript to make it more dynamic and user-friendly. We estimated the hypothetical coverage of primers conventionally used to study the diversity of photosynthetic microeukaryotes and cyanobacteria^44,57^ by performing an *in silico* PCR amplification.

### Illustration of the application of µgreen-db for studying complex soil phototrophic microbial communities

#### Soil sampling, experimental design

Soil samples were taken from the top 10 cm of a luvisol with a decarbonated sandy A horizon (pH = 8.2, C_org_ = 11.5 g kg^−1^, N_tot_ = 0.83 g kg^−1^) located in the north of Paris and used for conventional cropping, with a wheat/maize rotation. Soil was sampled and incubated either under a 16 h light/24 h photoperiod or continuous dark conditions, as described previously^76^, to obtain contrasted phototrophic microbial communities. Briefly, after sieving the soil at 5 mm and homogenising it, 6 microcosms were set up by placing 400 g of fresh soil weighed at 80% of its water-holding capacity in 0.825-dm^3^ glass jars. Three microcosms were coated with aluminium foil to prevent the development of phototrophic organisms (dark condition), and three microcosms were conditioned under a day/night cycle (light condition) consisting of a 16 h light/24 h photoperiod, using LED lighting with an intensity of around 200 μmol photons m^−2^ s^−1^ in the visible range to promote the growth of the native phototrophic organisms^76^. After 40 days of incubation at 20 °C with regular monitoring of soil moisture, one soil aliquot was sampled from each of the six microcosms and stored at −40 °C before DNA extraction.

#### Soil microbial DNA extraction, 23S rRNA gene amplification and Illumina sequencing

Microbial DNA was extracted and purified from 1 g of each soil sampled, using the GnSGII procedure described previously^77^. Crude DNA extracts were quantified by agarose gel electrophoresis and then purified using a GENECLEAN turbo kit (MpBiomedical), and quantified using a QuantiFluor staining kit (Promega) prior to further investigation.

A 23S rRNA gene fragment targeting the V5 domain to characterise photosynthetic microeukaryote and cyanobacterial diversity was amplified using the primers p23SrV_f1 (5′GGACAGAAAGACCCTATGAA3′) and p23SrV_r1 (5′TCAGCCTGTTATCCCTAGAG3′)^44^. Amplifications were carried out in a total volume of 25 μl composed of 5 μl of DNA (10 ng), 10 μl of buffer solution 10x containing 20 mM MgSO_4_ (Promega), 0.4 μl of dNTPs (25 mM, DNTPack 250U Roche), 2 μl (10 μM, Eurogentec) of each primer, 0.5 μl of Taq polymerase (5U/μl Taq PFU, Promega), 1.25 μl of T4 gene 32 (500 μg/mL, MP Biomedical) and 11.35 μl of mili-Q water. PCR1 conditions were as follows: 2 min at 94 °C, followed by 35 cycles of 45 s at 94 °C, 45 s at 63 °C, and 1 min at 72 °C, and final elongation for 10 min at 72 °C. After purification using a MinElute PCR purification kit (Qiagen), the purified PCR products were used as a matrix for a second PCR of seven cycles under similar PCR conditions (10 ng of DNA were used for a 25 µl of PCR mix), using fusion primers (‘p23SrV_f1/MID,’ ‘p23SrV_r1/MID). At the end of the seven cycles, the PCR products were purified using a MinElute PCR purification kit (Qiagen), and quantified using a QuantiFluor staining kit (Promega). For all libraries, an equimolar mix was obtained by pooling equal amounts from each of the 6 samples. The mix was then cleaned using the Agencourt AMPure XP system (Beckman Coulter Genomics). TE buffer (100 µl) (Roche) was used for elution. Sequencing was then carried out on an Illumina MiSeq system (GenoScreen, France).

#### Bioinformatics sequence analysis

To perform the raw data analysis of the 23S plastid rDNA amplicons generated from the soil samples, we used the GnS-PIPE pipeline available at: https://zenodo.org/record/1123425#.W82vmDVR2OE^78^. The different steps were described previously^79^. After preprocessing, filtering and chimera checking, all samples were normalised at 31.650 sequences. Taxonomic affiliation was performed using μgreen-db and the USEARCH program (v6.0.307; www.drive5.com/usearch) with specific parameters (-maxhits 15, -maxaccepts 0, and maxrejects 0). For alpha diversity analysis, we calculated various indices (Chao1, Shannon, Simpson)^80^. A Shannon index-based measure of evenness was also calculated (corresponding to the Evenness column in Supplementary Table S1). The microbial DNA sequence datasets supporting the results provided in this article are available at the EBI ENA under accession No. PRJEB30252.

To access the putative number of amplifications and the coverage of the different taxa, we performed an *in silico* PCR from μgreen-db. We used mothur software (v.1.40.5) with the *pcr.seqs* command and allowed zero mismatch between each of the primer pairs. Graphic representations were produced using custom scripts based on Highcharts facilities (http://www.highcharts.com/).

## Supplementary information

Supplementary Figure S2

Supplementary Information

Supplementary Tables

Supplementary Figure S1

## Data Availability

µgreen-db is available in flat files at http://microgreen-23sdatabase.ea.inra.fr and Zenodo repository (https://zenodo.org/record/3385760#.XW-NptPVLUI). The microbial DNA sequencing datasets supporting the results provided in this article are available at the EBI ENA under accession number PRJEB30252.
