# Supplementary material for: µgreen-db: a reference database for the 23S rRNA gene of eukaryotic plastids and cyanobacteria
Source: Sci Rep. 2020 Apr 3;10:5915. doi: 10.1038/s41598-020-62555-1 (PMC7125122; doi:10.1038/s41598-020-62555-1)
Supplement: Supplementary file 1 — Supplementary Figure S2 [file 41598_2020_62555_MOESM1_ESM.pdf]

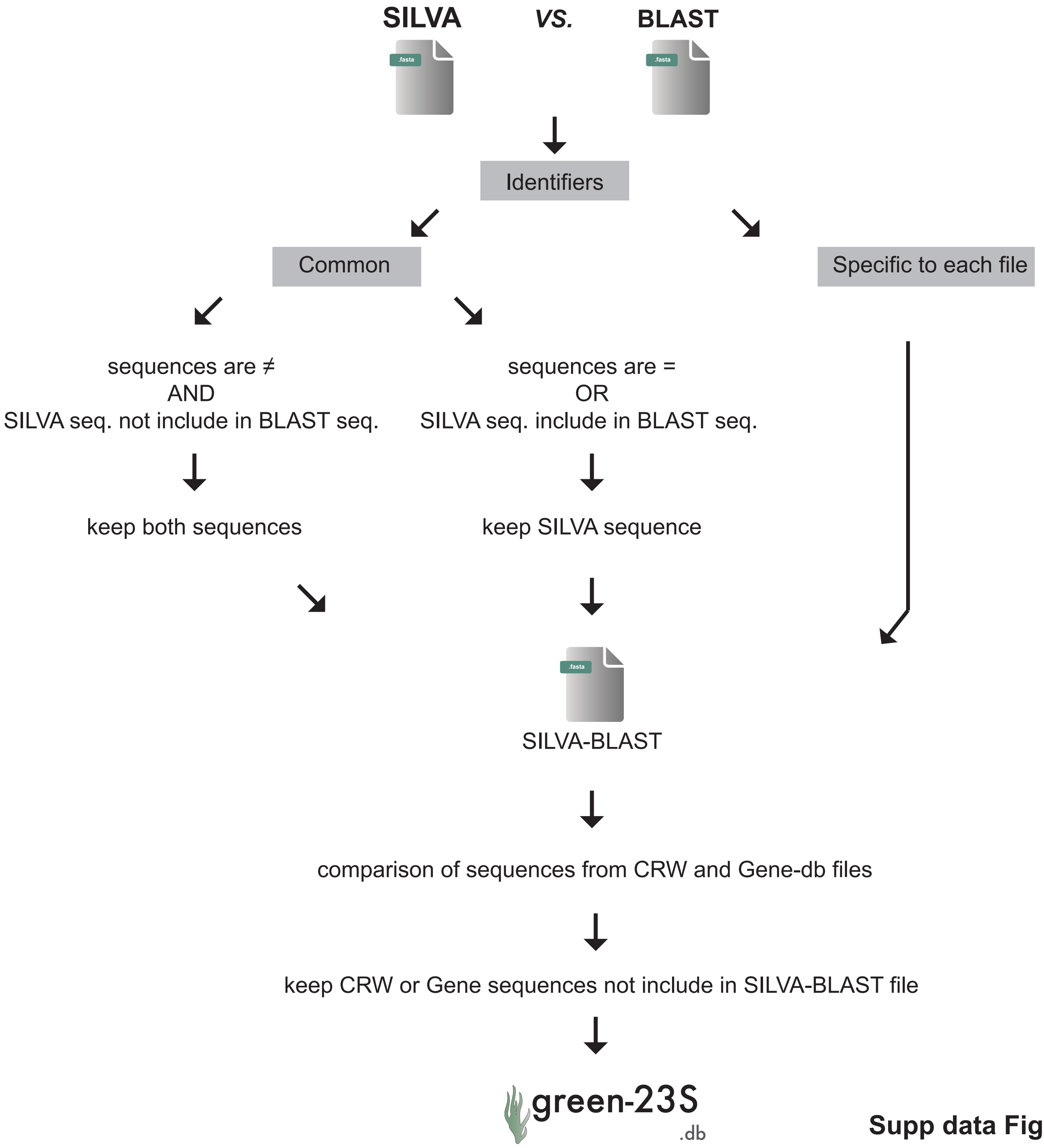

Supp data Figure 2
