# Supplementary material for: µgreen-db: a reference database for the 23S rRNA gene of eukaryotic plastids and cyanobacteria
Source: Sci Rep. 2020 Apr 3;10:5915. doi: 10.1038/s41598-020-62555-1 (PMC7125122; doi:10.1038/s41598-020-62555-1)
Supplement: Supplementary file 2 — Supplementary Information [file 41598_2020_62555_MOESM2_ESM.docx]

**µgreen-db: a reference database for the 23S rRNA gene of eukaryotic plastids and cyanobacteria**

Christophe Djemiel^1^, Damien Plassard^2^, Sébastien Terrat^1^, Olivier Crouzet^3^, Joana Sauze^4^, Samuel Mondy^1^, Virginie Nowak^1^, Lisa Wingate^4^, Jérôme Ogée^4^, Pierre-Alain Maron^1🟏^

*^1^ Agroécologie, AgroSup Dijon, INRA, Univ. Bourgogne Franche-Comté, Dijon, France*

*^2^ Plateforme GenomEast, IGBMC, CNRS UMR7104, Illkirch, France*

*^3^ Univ. Paris Saclay, AgroParisTech, UMR ECOSYS, INRA, F-78206 Versailles, France*

*^4^ INRA, Bordeaux Science Agro, UMR 1391 ISPA, 33140 Villenave d’Ornon, France*

^🟏^ Corresponding author: pierre-alain.maron@inra.fr

Phone: +33 (0)380 69 34 46

Fax: +33 (0)380 693 224

Address: UMR Agroécologie, 17 rue de Sully, 21065 Dijon, France

**Details on the procedure to retrieve, filter and construct the µgreen-db**

Following are some of the main elements for the retrieval, filtering and construction of the µgreen database. It must be emphasized that all information described below was valid at the time of our analyzes (2016) and it may no longer be valid now.

**SILVA (r123)**

Retrieval of plastidial 23S rDNA sequences (completes, incompletes, ‘possible rRNA’) for Cyanobacteria, algae and bryophytes.

**NCBI (Gene database)**

Keyword used to retrieve the plastidial 23S rDNA sequences

- Chloroplast large subunit ribosomal RNA

- rrl

- rnl

- 23S chlorophyta

- 23S ribosomal chlorophyta

- 23S ribosomal rhodophyta

- 23S ribosomal zygnematophyceae

- 23S ribosomal klebsormidiophyceae

- 23S ribosomal glaucophyta

- 23S ribosomal rhizaria

- 23S ribosomal euglenozoa

- 23S ribosomal cryptophyta

- 23S ribosomal haptophyta

- 23S ribosomal heterokonta

- large subunit ribosomal RNA

- 23S RNA NOT embryophyta

- 23S ribosomal marchiantophyta

- 23S ribosomal anthocerotophyta

- 23S ribosomal bryophyta

For the cyanobacteria we used the following query (23s ribosomal rna[All Fields] AND ("Cyanobacteria"[Organism] OR cyanobacteria[All Fields])

**The University of Texas at Austin**

The file was download from <http://www.rna.ccbb.utexas.edu/DAT/3C/Alignment/>.

We removed all the sequences that belonged to an organism not interesting to us but also the sequences having a too short length.

**BLAST (nr/nt database with default parameters)**

Blast1 : Marsupella emarginata

Blast2 : Takakia lepidozioides

Blast3 : Weimouthia cochlearifilia

Blast4 : Rhizogonium paramattense

Blast5 : Ptychomitrium incurvum

Blast6 : Targionia hypophylla

Blast7 : Verdigellas peltata

Blast8 : Cryptomonas okgeum

Blast9 : Porphyridium aerugineum

Blast10 : Ankyra judayi

Blast11 : Chlamydomonas iyengarii

Blast12 : Cymbomonas tetramitiformis M1669

Blast13 : Chroococcidiopsis clone NA2_6

Blast14 : Klebsormidium flaccidum

Blast15 : Cosmarium undulatum

Blast16 : Chaetosphaeridium globosum SAG 26_98

Blast17 : Euastrum substellatum svck364

Blast18 : Fottea pyrenoidosa

Blast19 : Pabia signiensis

Blast20 : Ignatius tetrasporus

Blast21 : Trebouxia sp. W0131

Blast22 : Dicloster acuatus

Blast23 : Tydemania expeditionis FL1151

Blast24 : Floydiella terrestris

Blast25 : Ulva fasciata

Blast26 : Cyanidioschyzon merolae

Blast27 : Euglenaformis proxima

Blast28 : Monomorphina pseudonordstedti FRC112909E

**BLAST (nr/nt database with max.hits = 1000)**

Blast1 : Marsupella emarginata

Blast2 : Takakia lepidozioides

Blast3 : Weymouthia cochlearifolia

Blast4 : Rhizogonium paramattense

Blast5 : Verdigellas peltata

Blast6 : Cryptomonas okgeum

Blast7 : Porphyridium aerugineum

Blast8 : Ankyra judayi

Blast9 : Klebsormidium flaccidum

Blast10 : Euastrum substellatum svck364

Blast11 : Euglenaformis proxima

Blast12 : Nodularia spumigena CCY9414

Blast13 : Hapalosiphon MRB220

Blast14 : Chamaesiphon minutus

Blast15 : Chlorotetraedron incus

Blast16 : Hafniomonas laevis

**Recovery of 23S sequences from whole genomes.**

**Concatenation of all data by removing the redundant sequences into a final file.**

**Verification of the secondary structure of the *genera* not found in the final file from the SILVA-Blast sequences by Infernal, of which we have a doubt.**

**Recovery of sequences obtained by Blast on WGS database.**

**Taxonomy verification**

We check all accession numbers to update when that was necessary.

From all accession numbers we retrieve the NCBI taxon ID (from ncbi ftp) and then the lineages with the following command lines.

taxonkit lineage --nodes-file taxdump/nodes.dmp --names-file taxdump/names.dmp ids > ids.lineage

taxonkit reformat --lineage-field 2 ids.lineage --nodes-file taxdump/nodes.dmp --names-file taxdump/names.dmp > ids.lineage.good

**Formatted files for metabarcoding data analysis**

BIOCOM-PIPE format

Example: : Eukaryota(domain);Stramenopiles(supergroup);Ochrophyta(phylum);Eustigmatophyceae(class);Eustigmatophyceae_X(order);Eustigmatophyceae_XX(family);Nannochloropsis(genus);Nannochloropsis_oculata

Mothur format

Example: d__Eukaryota;p__Ochrophyta;c__Eustigmatophyceae;o__Eustigmatophyceae_X;f__Eustigmatophyceae_XX;g__Nannochloropsis;s__Nannochloropsis_oculata;
