# Supplementary figures and images for: µgreen-db: a reference database for the 23S rRNA gene of eukaryotic plastids and cyanobacteria
Source: Sci Rep. 2020 Apr 3;10:5915. doi: 10.1038/s41598-020-62555-1 (PMC7125122; doi:10.1038/s41598-020-62555-1)

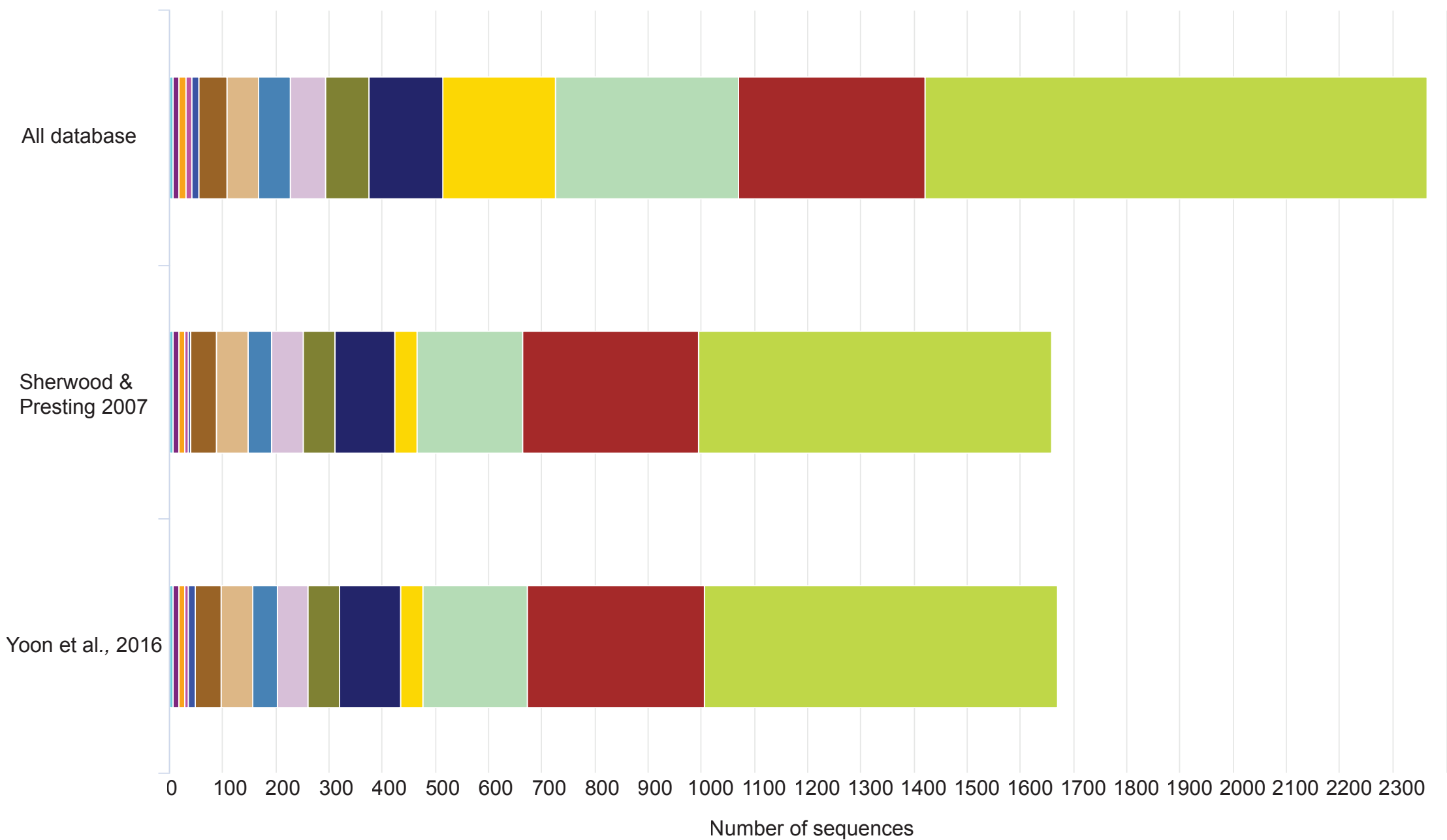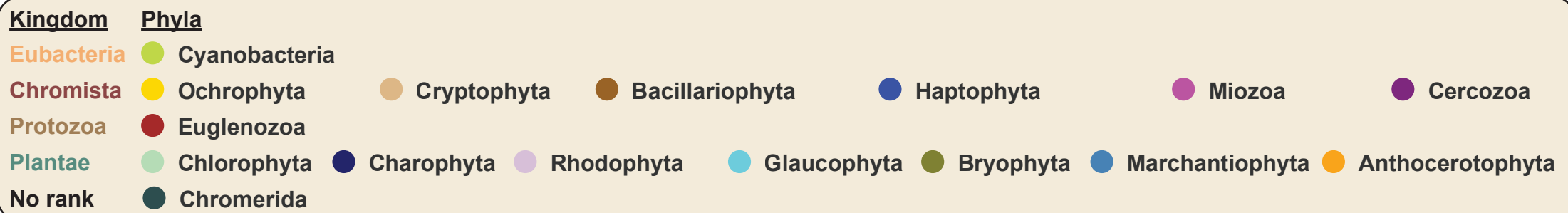

Supp data Figure 1

Supplement: Supplementary file 4 — Supplementary Figure S1 [file 41598_2020_62555_MOESM4_ESM.pdf]
